# Probiotic lactic acid bacteria as a means of preventing in vitro urinary catheter colonization and biofilm formation

**DOI:** 10.1186/s42506-022-00124-2

**Published:** 2023-01-03

**Authors:** Mohamed Anwar Mahgoub, Aleya Abdel Gawad Abbass, Amani Farouk Abaza, Mohamed Shafik Shoukry

**Affiliations:** 1 Department of Microbiology, High Institute of Public Health, Alexandria University, Alexandria, Egypt; 2 Department of Urology, Faculty of Medicine, Alexandria University, Alexandria, Egypt

**Keywords:** Probiotics, Catheter-associated urinary tract infections, Biofilm, Urinary catheter, Lactic acid bacteria, *Lactobacillus*

## Abstract

**Background:**

Catheter-associated urinary tract infections (CAUTIs) are the most common infections found in healthcare facilities. Urinary catheters predispose the development of CAUTIs by destroying natural barriers and providing a source for infection and biofilm formation (BF). This study aimed to evaluate probiotic lactic acid bacteria (LAB) as a means of preventing in vitro urinary catheter colonization and BF.

**Methods:**

Cross-sectional screening, followed by an experimental study, was conducted on 120 catheterized patients admitted to the urology department in a tertiary care hospital for 7 months. The isolated and identified uropathogens were tested for their antimicrobial susceptibility patterns by the disk diffusion method according to Clinical and Laboratory Standards Institute recommendations and examined for their ability to produce biofilms using a microtiter plate (MtP) assay. Five LAB (*Lactobacillus acidophilus (L. acidophilus)*, *Bifidobacterium bifidum (B. bifidum)*, *L. paracasei*, *L. pentosus*, and *L. plantarum*) were identified and examined for preventing in vitro colonization and BF of some isolated uropathogens on Foley urinary catheter surfaces.

**Results:**

Of the 120 samples collected, 32.5% were found to be associated with CAUTIs. Of isolated organisms, 74.4% were gram-negative bacilli, while gram-positive cocci represented 14%, and only 11.6% were of the *Candida* species. About two-thirds of isolated uropathogens were biofilm formers. All five probiotic strains had inhibitory effects on the growth of all the uropathogens tested but with varying intensities according to the duration of application after 2, 4, and 6 days.

**Conclusions:**

The prevalence of CAUTIs was high, and the predominant bacterial isolates were gram-negative bacilli. Many of the studied uropathogens were biofilm formers. The bacterial isolates had a higher prevalence of resistance to commonly prescribed antimicrobial agents. Probiotics have the potential to prevent in vitro urinary catheter colonization and inhibit BF. Pre-coating urinary catheters with probiotics is recommended after ensuring the safety of probiotics’ use in vivo by carrying out further large-scale studies.

**Supplementary Information:**

The online version contains supplementary material available at 10.1186/s42506-022-00124-2.

## Introduction

Urinary tract infections (UTIs) account for at least 40% of all healthcare-associated infections (HCAIs), and most cases are associated with catheters [1].

According to *the National Healthcare Safety Network* in 2019, the definition of catheter-associated urinary tract infection (CAUTI) includes indwelling urinary catheters in place for ˃ 2 calendar days and at least one of the following signs or symptoms: fever (> 38.0 °C), suprapubic tenderness, costovertebral angle pain, or tenderness. The definition also includes urine cultures with no more than two species of organisms, at least one of which is bacteria of ≥ 10^5^ CFU/mL [2].

Few accessible data have been reported from Egypt regarding CAUTI and its risk factors [3]. In a study conducted in a large hospital in Alexandria, Egypt, there was an overall rate of 15.7 CAUTI per 1000 catheter days [4]. The overall bacterial CAUTI incidence rate was found to be 11% in another study conducted in the intensive care unit of Assiut University Hospital, Egypt [3].

Most CAUTIs involve resistant bacteria from catheter-associated biofilm formation (BF). Therefore, routine surveillance of antimicrobial resistance and BF is necessary in all cases of UTI to ensure the proper management of patients [5].

A very promising approach for the control of biofilm-associated uropathogens is the use of probiotics, which are defined as “live organisms which when administered in adequate amounts, confer a health benefit on the host.” [6] Probiotics could be used to colonize hard surfaces to counteract the proliferation of other bacterial species [7].

Laboratory and clinical studies on probiotic bacteria have opened up an important research area with a growing number of experiments and trials [8]. Although much has been reported about the possibility of probiotics enhancing host health, there is little information about the actual effects of probiotics on uropathogens [9]. Given the enormous burden on patients, as well as the scientific and economic problems caused by recurrent UTIs, the investigation of probiotics is of potentially crucial importance for both patient benefit and clinical science [8]. This study aimed to evaluate probiotic lactic acid bacteria (LAB) as a means of preventing in vitro urinary catheter colonization and BF.

## Methods

### Study design

- First: A cross-sectional study to determine the bacteriological profile of CAUTIs
- Second: An experimental study to evaluate the role of LAB in preventing urinary catheters’ colonization and BF

### Sample size

Based on a previous study, 8.2% of hospitalized patients had a CAUTI [10], using a margin of error of 5%, alpha error = 0.05, and the minimum required sample size is 116 subjects. The sample size was calculated using Epi Info 7 software.

The study was conducted over 7 months from January to July 2019. It involved 120 urine samples randomly collected from adults with indwelling Foley urinary catheters, who were admitted to the Urology Department at the Alexandria Main University Hospital (AMUH), Egypt.

### Sample collection

The catheter tubing distal to the puncture site was clamped for 15–30 min to allow urine to fill the tubing. Then, the catheter port was disinfected using 70.0% ethanol. Urine samples were obtained by inserting sterile syringes aseptically into the catheter port and then transferred into labeled screw-capped sterile containers [11]. All the collected samples were transported within 2 h in an ice box to the Microbiology Laboratory of the High Institute of Public Health (HIPH) for processing.

### Sample processing

#### Isolation and antimicrobial susceptibility testing of uropathogens

A standard loop was inserted into the well-mixed urine and then spread over the surface of each of the blood, MacConkey and Sabouraud’s dextrose agar (SDA) plates. Blood and MacConkey agar plates were incubated at 37 °C for 24 h, while SDA plates were incubated at 25 °C for 48 h and up to 10 days [12]. The Quebec Colony Counter was used for counting the colonies. Plates yielded ≥ 10^5^ CFU/mL of one or maximally two organisms were considered positive for a UTI [13].

After incubation, the identification of isolated colonies was performed according to standard microbiological methods [12]. All bacterial isolates were subjected to antibiotic susceptibility testing by the disk diffusion method, [14] according to the Clinical and Laboratory Standards Institute recommendations [15].

#### Microtiter plate method for the detection of BFA

The biofilm-forming ability (BFA) of the identified uropathogens was tested by the MtP method according to Stepanović et al [16]. The experiments were performed in triplicate, and the results were averaged. Three colonies of each uropathogen (isolated from an overnight culture on Mueller-Hinton agar plates) were inoculated in 3 mL of tryptic soy broth with 1% glucose (TSBglu) and incubated for 24 h at 37 °C. The cultures were adjusted to a turbidity of 0.5-McFarland standards using phosphate-buffered saline (PBS) and diluted 1 in 100 with fresh TSBglu medium. Individual wells of sterile, 96-well MtP, were filled with 200 μL of the diluted cultures; negative control wells contained uninoculated sterile broth only. After incubation (24 h at 37 °C), the contents of each well were removed by gentle tapping, and the wells were washed three times with 300 μL of sterile PBS. The plates were drained in an inverted position. The biofilms formed by bacteria adherent to the wells were heat fixed by exposure to hot air at 60 °C for 60 min and then stained with 150-μL crystal violet (2% w/v) for 15 min. Excess staining was removed by using running tap water, and the plates were kept for drying.

The optical density (OD) of each well was obtained using a microplate enzyme-linked immunosorbent assay reader (Dialab ELX800G, Vienna, Austria) at wavelength 630 nm. The experiment was performed in triplicate, and the results were averaged. The cutoff OD (ODc) was calculated as three standard deviations above the mean OD of the negative control. The isolates were classified as follows:

- OD ≤ ODc — non-biofilm former
- ODc ˂ OD ≤ 2 × ODc — weak biofilm former
- 2 × ODc ˂ OD ≤ 4 × ODc — moderate biofilm maker
- 4 × ODc ˂ OD — strong biofilm former

#### Immobilization of probiotic strains on catheter pieces [17]

Five probiotic strains (*L. acidophilus*, *L. plantarum*, *L. paracasei*, *L. pentosus*, and *B. bifidum*) which showed the best probiotic properties (auto-aggregation, co-aggregation, safety, and hemolytic activities) were selected from a previously conducted PhD study at the HIPH.

Five 10 mL De Man Rogosa Sharpe (MRS) broth tubes containing *probiotic* cells were used. Each tube was inoculated with 10^6^ CFU of different probiotic strains (*L. acidophilus*, *L. plantarum*, *L. paraquisi*, *L. pentosus*, and *B. bifidum*). All tubes were incubated aerobically for 48 h at 37 °C. The broth tubes were centrifuged at 5000 rpm for 15 min. From each tube, the supernatant was discarded, while the pellet of cells was added to a beaker containing 2% (w/v) sodium alginate solution. Twelve sterile Foley catheters were purchased from the market, and each catheter was divided into 10 equal pieces. The catheter pieces were then introduced into the mixture for 1 h (24 catheter pieces in each beaker). Each of the 24 catheter pieces was extracted and immersed in a separate beaker containing 2% calcium chloride solution and incubated aerobically for 24 h to allow the formation of a gel.

From each beaker containing immobilized *probiotic* cells, three catheter pieces were immersed in broth cultures of each of the selected eight urine isolates (two *Klebsiella pneumoniae* (*K. pneumoniae*), two *Escherichia coli* (*E. coli*), one *Proteus mirabilis* (*P. mirabilis*), one *Pseudomonas aeruginosa* (*P. aeruginosa)*, one *Enterococcus* spp*.*, and one *Candida* spp.) in separate sterile bottles and were allowed to stand for 6 days at 37 °C. Biofilm formation on the catheter sections was evaluated by a viable cell count procedure after 2, 4, and 6 days.

On each day of counting, a catheter piece was picked up from the various cultures and rinsed with sterile distilled water to remove unattached cells. Then, the attached cells were gently scraped off from both the outer and luminal surfaces of the catheters using a wire loop and introduced into sterile beakers containing 10 ml of PBS. The cells in the biofilm were dispersed using a magnetic stirrer, and a loopful of the broth was spread on blood, MacConkey, and MRS agar plates for viable counts. Sabouraud dextrose agar was used instead of a MacConkey plate with *Candida* spp. Blood and MacConkey agar plates were incubated at 37 °C for 24 h, while MRS agar and SDA were incubated for 48 h (at 35 °C for MRS and 25 °C for SDA).

Colonies were counted using a Quebec Counter and expressed as follows:

- No colonies/mL
- Less than 50 CFU/mL
- 50: 100 CFU/mL
- More than 100 CFU/mL

### Statistical analysis

Data were analyzed using the Statistical Package for the Social Sciences software version 20. Data were presented as numbers and percentages for categorical variables and as means and standard deviations (SD) for continuous variables. All results were interpreted at a 5% level of significance.

## Results

The present study was conducted on 120 catheterized patients (69 males and 51 females) who were admitted to the urology department of a tertiary care hospital. Demographic and some clinical data are illustrated in Table 1.

**Table 1:** Distribution of the 120 studied catheterized patients according to their demographic and some clinical data in AMUH, 2019

Catheterized patients (***n*** = 120)	No.	%
**Sex**		
Male	69	57.5
Female	51	42.5
**Age (years)**		
< 40	03	2.5
40–60	24	20.0
> 60	93	77.5
Min–max^a^	27–92	
Mean ± SD^b^	68.28 ± 10.93	
**Reasons for hospitalization**		
Prostatic surgery	50	41.7
Renal and ureteric surgery	63	52.5
Others	07	5.8
**Signs and symptoms**^d^		
Fever	12	10.0
Suprapubic tenderness	15	12.5
Costovertebral angle pain	22	18.3
**Past admission**		
Yes	36	30.0
**Past surgery**		
**Yes**	23	19.2
**Comorbid conditions**^c^		
Diabetes mellitus	27	22.5
Hypertension	33	27.5
Other	02	1.7
**Duration of catheterization (days)**		
Min–max^a^	1–8	
Mean ± SD^b^	3.52 ± 1.62	
**Duration of antibiotic intake (days)**		
Min–max^a^	1–7	
Mean ± SD^b^	2.76 ± 1.5	

^a^Minimum–maximum. ^b^Standard deviations. ^c^Each patient might have had more than one comorbid condition. ^d^Each patient might have one or more signs and symptoms

Table 2 shows that of the 67 patients who had been catheterized for less than or equal to 2 days, only 8 (20.5%) patients had CAUTIs, while of the 53 patients who had been catheterized for more than 2 days, 31 (79.5%) had CAUTIs. The difference between these figures was found to be statistically significant (*P*-value < 0.001).

**Table 2:** Relationship between duration of catheterization and CAUTI among the 120 studied catheterized patients in AMUH, 2019

Catheterization days	CAUTI				Total (***n*** = 120)		***P***
	No (***n*** = 81)		Yes (***n*** = 39)				
	No.	%	No.	%	No.	%	
≤ 2	59	72.8	8	20.5	67	55.8	< 0.001*
>2	22	27.2	31	79.5	53	44.2	
Mean ± SD	2.22 ± 1.14		3.56 ± 1.50		2.66 ± 1.41		< 0.001*
Median (Q1–Q3)	67.00 (59.00–74.00)		71.00 (65.00–81.00)		68.50 (61.00–75.75)		< 0.001*

*p p*-values for chi-square test. *Statistically significant at *p* ≤ 0.05

**Fig. 1:**
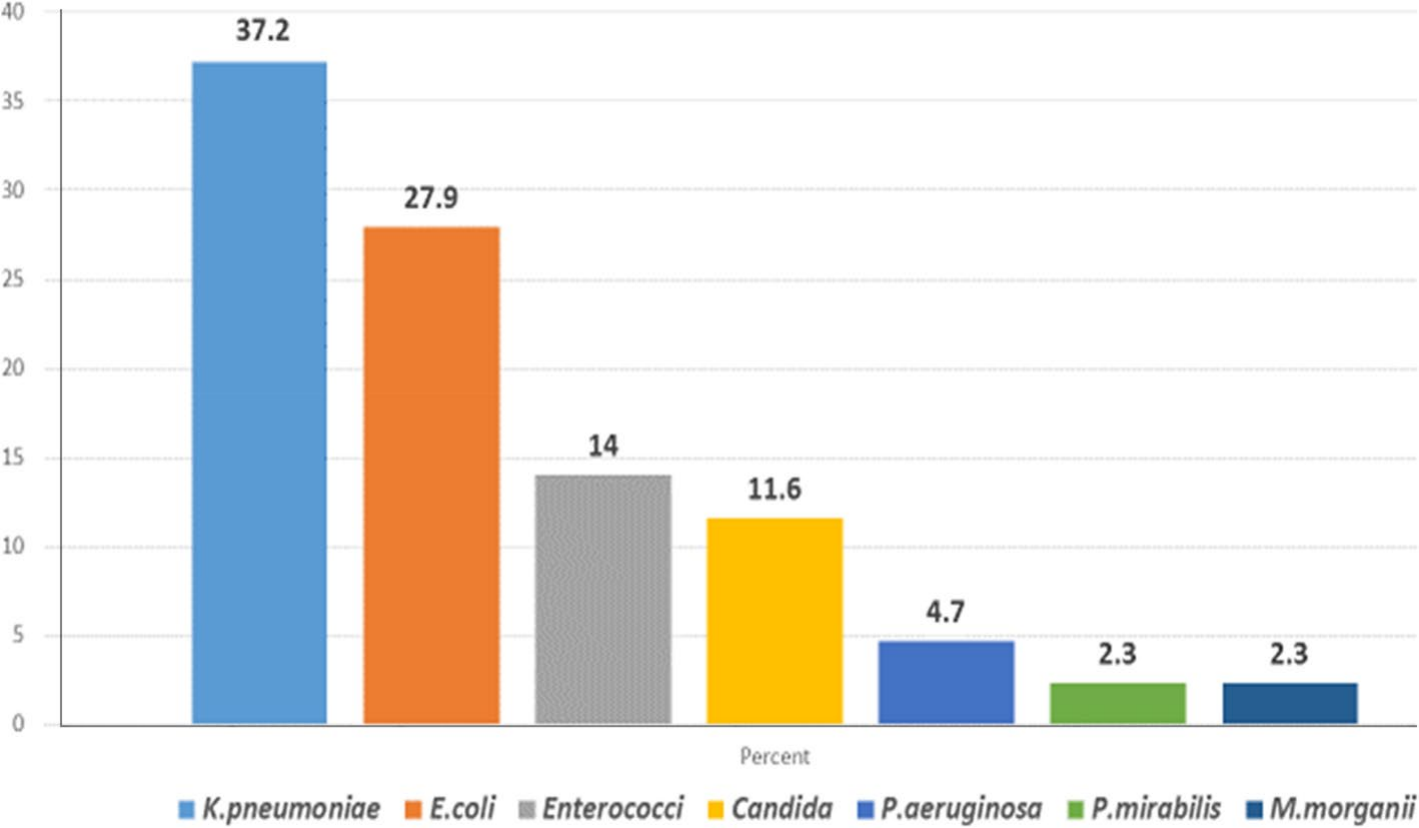
Distribution of 43 uropathogenic isolates from the studied 120 catheterized patients in the Alexandria Main University Hospital (AMUH), Egypt, 2019

Figure 1 shows that out of the 43 bacterial and yeast fungi isolates, 32 (74.4%) were gram-negative bacilli, 6 (14.0%) were gram-positive cocci, and only 5 (11.6%) were fungi. The most common microbial isolate was *K. pneumoniae* (37.2%), followed by *E. coli* (27.9%).

Table 3 shows the antimicrobial susceptibility percentages of the 38 identified uropathogenic bacterial isolates to different groups of antibiotics. None of the *K. pneumoniae* isolates was susceptible to nitrofurantoin or fosfomycin, but half of the *K. pneumoniae* isolates (50.0%) were susceptible to imipenem. The highest susceptibility percentage of *E. coli* was recorded for imipenem (66.7%), whereas the lowest susceptibility percentages were detected for ampicillin, cephazolin, ceftriaxone, ceftazidime, ciprofloxacin, and gentamycin (8.3%) each.

**Table 3:** Antimicrobial susceptibility percentages of the 38 identified uropathogenic bacterial isolates recovered from the 120 studied patients in AMUH, 2019

Group	Bacterial isolates (38)	Penicillins	β-Lactam/β-lactamase inhibitor combination		Cephems (parenteral)			Carbapenems	Fluoroquinolones		Aminoglycosides	Folate pathway antagonists	Nitrofurans	Fosfomycin	Oxazolidinone
		AMP	AMC	TZP	CZ	CRO	CAZ	IPM	CIP	LE	GN	SXT	NI	FOS	LZD
**Gram-positive cocci**	***Enterococcus*** **spp. (6)**	83.3	83.3	83.3	NA	NA	NA	83.3	0.0	0.0	NA	NA	83.3	100	100.0
**Gram-negative bacilli**	***K. pneumoniae*** **(16)**	NA	43.8	25.0	6.3	18.8	18.8	50.0	12.5	12.5	31.3	18.8	0.0	0.0	NA
	***E. coli*** **(12)**	8.3	33.3	41.7	8.3	8.3	8.3	66.7	8.33	16.7	8.3	16.7	50.0	58.3	NA
	***P. aeruginosa*** **(2)**	NA	NA	50.0	NA	NA	50.0	50.0	50.0	50.0	50.0	NA	NA	0.0	NA
	***P. mirabilis*** **(1)**	100.0	100.0	100.0	100.0	100.0	100.0	100.0	0.0	0.0	0.0	0.0	NA	100.0	NA
	***Morganella morganii (1)***	NA	NA	100.0	NA	100.0	0.0	0.0	0.0	0.0	0.0	0.0	NA	0.0	NA

*NA* Not applicable, *AMP* Ampicillin, *AMC* Amoxicillin-clavulanate, *CZ* Cephazolin, *CRO* ceftriaxone, *CAZ* Ceftazidime, *TZP* Piperacillin-tazobactam, *IPM* Imipenem, *CIP* Ciprofloxacin, *LE* Levofloxacin, *GN* Gentamicin, *NI* Nitrofurantoin, *FOS* Fosfomycin, *LZD* Linezolid, *SXT* Trimethoprim-sulfamethoxazole

Table 4 shows the distribution of 27 multidrug-resistant (MDR) uropathogenic bacterial isolates. It is noted from Table 5 that about two-thirds (67.44%) of all uropathogenic isolates were positive for BFA. The BFA of the isolates was classified based on the measured ODs into weak, moderate, and strong.

**Table 4:** Distribution of the 27 MDR^*^ uropathogenic bacterial isolates from the 120 studied catheterized patients in AMUH, 2019

Group	Bacterial isolates (***n*** = 38)	MDR^a^	
		No.	%
**Gram-positive cocci**	*Enterococcus* spp. (6)	1	16.8
**Gram-negative bacilli**	*K. pneumoniae* (16)	13	82.3
	*E. coli* (12)	11	91.7
	*P. aeruginosa* (2)	1	50.0
	*P. mirabilis* (1)	0	0.0
	*M. morganii (1)*	1	100.0
	**Total**	**27**	**71.1**

^a^MDR uropathogens were those isolates that showed resistance to at least one agent in three or more of the antimicrobial categories

**Table 5:** BFA of the 43 identified uropathogenic isolates from the 120 studied catheterized patients in AMUH, 2019

Identified uropathogenic isolates		BFA		
			No.	%
**Gram-positive cocci**	***Enterococcus*** **spp.**	Negative	1	16.7
		Positive	5	83.3
**Gram-negative bacilli**	***E. coli***	Negative	8	66.7
		Positive	4	33.3
	***K. pneumoniae***	Negative	2	12.5
		Positive	14	87.5
	***P. mirabilis***	Negative	0	0.0
		Positive	1	100.0
	***M. morganii***	Negative	1	100.0
		Positive	0	0.0
	***P. aeruginosa***	Negative	0	0.0
		Positive	2	100.0
**Fungi**	***Candida*** **spp.**	Negative	2	40.0
		Positive	3	60.0
***Total***		Negative	14	32.6
		Positive	29	67.4

### The effect of different probiotic strains on selected identified uropathogenic isolates

All five probiotic strains converted the two selected *E. coli* strains and only *K. pneumoniae* (strain two) from biofilm producers (BPs) to non-BPs, while *K. pneumoniae* (strain one), *P. mirabilis,* and *P. aeruginosa* remained capable of BF. Only *L. acidophilus* and *B. bifidum* converted the selected *Enterococcus* spp. BP to a non-BP, while all probiotics except *L. plantarum* had the same effect on selected *Candida* spp.

All five tested probiotics changed *K. pneumoniae* strain (one) from strong-to-moderate BP. *B. bifidum*, *L. pentosus*, and *L. plantarum* converted the selected moderate BP *P. mirabilis* isolates to weak BP, while only *B. bifidum* and *L. plantarum* converted the selected moderate BP *P. aeruginosa* to weak BP. *L. paracasei*, and *L. plantarum* converted the selected moderate *Enterococcus* spp. to weak BP.

### Results of the in vitro study

*L. acidophilus*, *B. bifidum*, and *L. pentosus* significantly decreased the counts of the selected uropathogens from ≥ 10^5^ to < 10^5^ CFU/mL after 4 and 6 days (*P* < 0.008), while *L. paracasei* and *L. plantarum* significantly decreased the counts to < 10^5^ CFU/mL after 2 (*P* < 0.031), 4 (*P* < 0.016 and <0.008, respectively), and 6 days (*P* = 0.008).

Only *L. acidophilus* and *L. pentosus* significantly decreased the counts of the selected uropathogens to < 10^3^ CFU/mL after 6 days (*P* < 0.031).

## Discussion

CAUTIs represent about 30–40% of all healthcare-associated infections [18]. It is considered a major source of healthcare-associated septicemia and related mortality in acute care hospitals. Urinary catheters increase the tendency to UTI by disrupting natural barriers and acting as surfaces for BF. Biofilm-forming pathogens represent a major problem in CAUTIs as they affect up to 75–80% of all infections [5].

In this study, CAUTIs represented 32.5% of total infections. This is somewhat higher than the previously published rates. In a study conducted in Egypt, the rate of CAUTI was found to be 11% [6], while Jiménez-Alcaide et al. reported in their study that the incidence of CAUTI was 8.2% [10]. Our higher results are probably because most patients enrolled in this study were elderly, and about half of them suffered from comorbid conditions that increase the possibility of development of CAUTIs [19]. At the same time, about 67.5% of the samples examined in the present study yielded nonsignificant microbial growth. This could be attributed to the fact that almost all enrolled patients were covered by broad-spectrum antibiotics (cephalosporins, quinolones, or both) and the short duration of catheterization (which ranged only from 1 to 8 days). The most important risk factor for the development of CAUTI is the duration of catheterization, as has been verified in several studies. Increased duration presumably increases the likelihood of microbes ascending to the bladder either around the catheter or through its lumen [19, 20]. This was in accord with our findings, where there was a significant association between the duration of catheterization and CAUTI.

According to the results of the present study, most CAUTIs showed monomicrobial growth (89.7%), while only four urine samples showed two isolated organisms with significant growth (10.3%). This concurred with other studies conducted by Béla Köves et al. and Aly et al. where about 90% of CAUTIs were monomicrobial in short-term catheterization [1, 3].

Many healthcare surveillance studies consistently identify *E. coli*, *Klebsiella* spp., and *Enterococci* spp. as the predominant uropathogens causing CAUTIs. These findings are in agreement with our results, where the most common microbial isolates were *K. pneumoniae* (37.21%), *E. coli* (27.91%), and *Enterococcus* spp. (14%) followed by *Candida* spp. (11.63%) and *P. aeruginosa* (4.7 %). In the present study, *K. pneumoniae* was the most commonly identified bacterium, although *E. coli* is primarily considered the most prevalent etiological agent for CAUTI. This finding may reflect the difference in bacterial populations according to different sites and indicates the role of the environment in the shaping of the bacterial population in each healthcare center [3].

Many bacterial uropathogens that were recovered in this study were found to be resistant to most of the tested antimicrobials. These findings are in line with prior studies which demonstrated that organisms recovered from hospitalized patients are often resistant to multiple antibiotics [21, 22]. The high rate of MDR uropathogens in the present study (71.1%) reflects the extensive use of antimicrobials in healthcare facilities. It has been reported by Exner et al. that high rates of multidrug resistance can be attributed to the ability of the organism to acquire resistance genes [23]. Although nearly all patients in this study were covered by third-generation cephalosporins and/or quinolone antibiotics, imipenem was the most effective drug against *K. pneumoniae* (50%) and *E. coli* (66.6%). Resistance to carbapenem over time is terrible and creates a risk for infected patients, being the only antimicrobial option for some MDR isolates.

The current study showed that 67.44% of the uropathogens had BFA, whereas 32.56% isolates were non-BPs. Similar findings have been reported by Sabir et al. (73.4%) [24], while only 46% of strains were in vitro positive for the biofilm production in a study conducted by Mahrajan et al. [25] These variations were attributed to the differences in types and virulence factors of isolates and duration of catheterization [24].

In the present study, only one-third of *E. coli* isolates (33.33%) had BFA, while the majority *of K. pneumoniae* isolates (87.5%) were BPs, and these findings were different from a study in which *E. coli* had a greater ability to form biofilm [26]. However, a study conducted by Ramos-Vivas et al. showed that 16% of *E. coli* strains and 73% of *K. pneumoniae* strains showed BF [27]. Another study conducted by Surgers et al. reported that *E. coli* and *K. pneumoniae* have large differences in the proportion of developing biofilm, with almost half of *E. coli* and 80% of *K. pneumoniae* producing biofilm [28].

In this study, pre-coating of the catheter surface with LAB reduced the attachment and growth of bacterial uropathogens. The effect was more pronounced against *E. coli* and *K. pneumoniae* (strain two), with a near-complete inhibition being achieved by the 6th day of co-culture by all probiotic strains. However, 6 days were not enough to clear *P. aeruginosa* by any of the tested probiotic strains. The results of this study strongly agree with those of Ifeoma and Jennifer, in which pre-coating of catheter surfaces with *L. acidophilus* before exposure to bacterial uropathogens significantly reduced the attachment of *E. coli* and *Klebsiella* to the catheter surfaces, while *P. aeruginosa* was not inhibited [29]. The results of the current study also agree with those of Maldonado et al., in which whole cells and acid supernatant of *L. fermentum* inhibited BF and growth of *Klebsiella* [30].

The results of this study showed that LAB had the potential to inhibit urinary catheter biofilms when applied to catheters, and the inhibitory action could be both by removal of the attachment surface for bacterial uropathogens and the production of antibacterial substances.

### Limitations of the study

Only hospitalized patients were included, and molecular identification of the biofilm was not performed.

## Conclusion

CAUTIs remain a serious problem in hospitals, and what complicates the situation is that most causative uropathogens are MDR and have BFA with few options for therapy. The prevalence of CAUTI was high, and there was a significant association between the duration of catheterization and CAUTI. The predominant bacterial isolates were gram-negative bacilli, mainly *K. pneumoniae* and *E. coli*. Many of the studied uropathogens were biofilm formers. In addition, bacterial isolates had a higher prevalence of resistance to commonly prescribed antimicrobial agents. Probiotics caused a significant reduction in uropathogenic counts after 4 and 6 days of application. *L. acidophilus* was found to have a significant inhibitory effect on BFA and colonization of tested uropathogens on Foley urinary catheters. This study puts forward an interesting intervention strategy that should be further evaluated in large-scale studies for in vivo use. Concerns that may come to mind in consideration of practical application are those relating to the blockage of the catheter by the coating as well as the possibility of infection by the LAB. These questions can be answered by carrying out further trials using experimental animals.

## Supplementary Information

**Additional file 1: Supplementary Tables 1.** Effect of different probiotic strains on BFA of selected uropathogenic isolates in AMUH, 2019. **Table 2.** Effect of *L. acidophilus* on the selected uropathogenic isolates in AMUH, 2019. **Table 3.** *L.acidophilus* effect on decreasing the counts of the eight selected uropathogens from ≥10^5^ CFU/mL to <10^5^ CFU/mL after 2, 4 and 6 days in AMUH, 2019.

## Data Availability

The datasets used and/or analyzed during the current study are available from the corresponding author on reasonable request.

## Abbreviations

AMUH: Alexandria Main University Hospital
*B. bifidum*: *Bifidobacterium bifidum*
BF: Biofilm formation
BFA: Biofilm-forming ability
BPs: Biofilm producers
CAUTIs: Catheter-associated urinary tract infections
CFU/mL: Colony-forming unit/milliliter
*E. coli*: *Escherichia coli*
HCAIs: Healthcare-associated infections
HIPH: High Institute of Public Health
*K. pneumoniae*: *Klebsiella pneumoniae*
*L. acidophilus*: *Lactobacillus acidophilus*
*L. paracasei*: *Lactobacillus paracasei*
*L. plantarum*: *Lactobacillus plantarum*
LAB: Lactic acid bacteria
MDR: Multidrug resistant
MRS: De Man, Rogosa, and Sharpe
MtP: Microtiter plate
OD: Optical density
OD_c_: Cutoff OD
*P. aeruginosa*: *Pseudomonas aeruginosa*
*P. mirabilis*: *Proteus mirabilis*
PBS: Phosphate-buffered saline
SDA: Sabouraud dextrose agar
UTIs: Urinary tract infections
