# Supplementary material for: Probiotic lactic acid bacteria as a means of preventing in vitro urinary catheter colonization and biofilm formation
Source: J Egypt Public Health Assoc. 2023 Jan 3;97:30. doi: 10.1186/s42506-022-00124-2 (PMC9807722; doi:10.1186/s42506-022-00124-2)
Supplement: Supplementary file 1 — Additional file 1: Supplementary Tables 1. Effect of different probiotic strains on BFA of selected uropathogenic isolates in AMUH, 2019. Table 2. Effect of L. acidophilus on the selected uropathogenic isolates in AMUH, 2019. Table 3. L.acidophilus effect on decreasing the counts of the eight selected uropathogens from ≥105 CFU/mL to <105 CFU/mL after 2, 4 and 6 days in AMUH, 2019. [file 42506_2022_124_MOESM1_ESM.docx]

**Table (1): Effect of different probiotic strains on BFA of selected uropathogenic isolates in AMUH, 2019.**

| **Selected uropathogenic strains** | **BFA without probiotic strains** | **BFA with probiotic strains** | | | | |  |  |
| --- | --- | --- | --- | --- | --- | --- | --- | --- |
|  |  | ***L.acidophilus*** | ***Bifidiobacterium*** | ***L.paracasei*** | ***L.pentosus*** | ***L.plantarum*** | ***Total present*** | ***% reduction*** |
| ***E.coli* (strain one)** | present | **Absent** | **Absent** | **Absent** | **Absent** | **Absent** | 0 | **100%** |
| ***E.coli* (strain two)** | present | **Absent** | **Absent** | **Absent** | **Absent** | **Absent** | 0 | **100%** |
| ***K. pneumoniae* (strain one)** | present | Present | Present | Present | present | present | 5 | **0.0%** |
| ***K. pneumoniae* (strain two)** | present | **Absent** | **Absent** | **Absent** | **Absent** | **Absent** | 0 | **100%** |
| ***P. mirabilis*** | present | Present | Present | Present | present | present | 5 | **0.0%** |
| ***P. aeruginosa*** | present | Present | Present | Present | present | present | 5 | **0.0%** |
| ***Enterococcus* spp.** | present | **Absent** | **Absent** | Present | present | present | 3 | **40.0%** |
| ***Candida* spp.** | present | **Absent** | **Absent** | **Absent** | **Absent** | present | 1 | **20.0%** |
| ***Total present*** | 8 | 3 | 3 | 4 | 4 | 5 |  | |
| ***% reduction*** |  | **62.5%** | **62.5%** | **50.0%** | **50.0%** | **37.5%** |  |  |

**Table (2): Effect of *L.acidophilus* on the selected uropathogenic isolates in AMUH, 2019.**

| **Selected uropathogenic strains** | **Count without *L.acidophilus*** | **Count with *L.acidophilus*** | | |
| --- | --- | --- | --- | --- |
|  |  | **After 2 days** | **After 4 days** | **After 6 days** |
| ***E.coli* (Strain one)** | +++ | ++ | + | + |
| ***E.coli* (Strain two)** | +++ | +++ | + | + |
| ***K. pneumoniae* ( Strain one)** | +++ | ++ | + | + |
| ***K. pneumoniae* (Strain two)** | +++ | ++ | + | + |
| ***P. mirabilis*** | +++ | ++ | ++ | ++ |
| ***P. aeruginosa*** | +++ | +++ | ++ | ++ |
| ***Enterococcus* spp.** | +++ | +++ | ++ | + |
| ***Candida* spp.** | +++ | + | + | + |

(+++) ≥ 10^5^ CFU/mL

(++) ≥10^3^ : <10^5^ CFU/mL

(+) <10^3^ CFU/mL

**Table (3): *L.acidophilus* effect on decreasing the counts of the eight selected uropathogens from ≥10^5^ CFU/mL to <10^5^ CFU/mL after 2, 4 and 6 days in AMUH, 2019.**

| **Uropathogens**  **Count**  **(CFU/ml)** | **Without *L.acidophilus*** | | **With  *L.acidophilus* after 2 days** | | **With  *L.acidophilus* after 4 days** | | **With  *L.acidophilus***  **after 6 days** | |
| --- | --- | --- | --- | --- | --- | --- | --- | --- |
| <10^5^ | 0 | 0.0 | 5 | 62.5 | 8 | 100.0 | 8 | 100.0 |
| ≥10^5^ | 8 | 100.0 | 3 | 37.5 | 0 | 0.0 | 0 | 0.0 |
| P |  | | 0.063 | | 0.008* | | 0.008* | |

*: Statistically significant at p ≤ 0.05
